# Analysis of LncRNA-mRNA Co-Expression Profiles in Patients With Polycystic Ovary Syndrome: A Pilot Study

**DOI:** 10.3389/fimmu.2021.669819

**Published:** 2021-04-14

**Authors:** Xiuhong Sun, Yishan Liu, Xinyu Gao, Mengxuan Du, Mengge Gao, Xingming Zhong, Xiangcai Wei

**Affiliations:** ^1^ Department of Reproductive Immunity, Guangdong Women and Children Hospital, Guangzhou Medical University, Guangzhou, China; ^2^ Department of Medical Ultrasonics, Guangzhou Women and Children’s Medical Center, Guangzhou Medical University, Guangzhou, China; ^3^ Department of Epidemiology, School of Medicine, Jinan University, Guangzhou, China; ^4^ Department of Reproductive Immunity, Family Planning Research Institute of Guangdong Province, Guangzhou, China

**Keywords:** polycystic ovary syndrome, long noncoding RNA, messenger RNA, peripheral blood, gene ontology, pathway analysis

## Abstract

**Purpose:**

This study aimed to investigate the profiles of messenger RNAs (mRNAs) and long noncoding RNAs (lncRNAs) in peripheral blood samples collected from polycystic ovary syndrome (PCOS) patients. In addition, an in-depth bioinformatics analysis regarding the lncRNA-mRNA co-expression network was performed.

**Methods:**

High-throughput sequencing was used to measure the profiles of mRNAs and lncRNAs expressed in the peripheral blood samples isolated from six patients (three patients with PCOS and three normal women). In addition, five differentially expressed lncRNAs were chosen to validate the results of high-throughput sequencing by quantitative RT-PCR (qRT-PCR). Furthermore, a lncRNA-mRNA co-expression network was constructed using the Cytoscape software.

**Results:**

A total of 14,276 differentially expressed mRNAs and 4,048 differentially expressed lncRNAs were obtained from the RNA-seq analysis of PCOS patients and healthy controls (adjusted q-value < 0.05, Fold change >2.0).The qRT-PCR results were consistent with the data obtained through high-throughput sequencing. Gene ontology (GO) and KEGG pathway analyses showed that the differentially expressed mRNAs were enriched in the chemokine signaling pathway. In addition, the analysis of the lncRNA-mRNA co-expression network of the chemokine signaling pathway showed the involvement of 6 mRNAs and 42 lncRNAs.

**Conclusion:**

Clusters of mRNAs and lncRNAs were aberrantly expressed in the peripheral blood of PCOS patients compared with the controls. In addition, several pairs of lncRNA-mRNAs in the chemokine signaling pathway may be related to PCOS genetically.

## Introduction

As one of the most common endocrinopathies in women of reproductive age, polycystic ovary syndrome (PCOS) is characterized by oligo-anovulation, hyperandrogenism, insulin resistance and increased risks of long-term endometrial cancer, metabolic syndrome (1), type 2 diabetes (T2D), and cardiovascular diseases (2). PCOS patients not only have pathological changes in the ovaries, leading to dysfunction, but also corresponding changes and even damage to their endometrial acceptance and embryo implantation. Although the etiology of PCOS remains unclear, most researchers believe that the causes of PCOS are multifactorial and genetic factors play pivotal roles in its pathogenesis and prognosis (3). In most PCOS studies, the samples were collected from experimental animals, human ovarian granulosa cells (4), whole ovaries (5), oocytes (6) and cumulus cells (7), which pay more emphasis on the local microenvironment of the ovary and revealed that many genes are associated with PCOS. However, as a systemic disease, PCOS can be investigated more comprehensively using peripheral blood samples.

In general, long noncoding RNAs (lncRNAs) are defined as RNA transcripts longer than 200 nucleotides that lack protein-coding capability (8). LncRNAs have been found to exist in a stable form and are protected from endogenous RNase activity in tissues and body fluids, such as urine and blood (9). The major function of lncRNAs is to regulate cell growth, proliferation, differentiation, and apoptosis (10, 11). In addition, recent studies have shown that lncRNAs may be associated with the pathogenesis of PCOS. For example, C-Terminal binding protein 1 antisense (CTBP1-AS) was a novel lncRNA found to regulate androgen receptor (AR) activity (12), while lncRNA SRA might act as an important mediator of adiposity-related processes in PCOS.

Co-expression analysis is widely used to elucidate the relationship between lncRNAs and messenger RNAs (mRNAs) (13). It can also be used to discover key lncRNAs and to understand their underlying mechanisms in important diseases including PCOS. Therefore, high-throughput sequencing was performed in this study to examine the profiles of lncRNA and mRNA expression in blood samples collected from PCOS patients and healthy controls.

## Materials and Methods

Patient characteristics and Samples From June 2019 to May 2020, 122 patients (63 patients with PCOS and 59 normal subjects) admitted to Guangdong Family Planning Institute of Science and Technology were included in this study. The protocol was approved by the Ethics Committee of Guangdong Family Planning Institute of Science and Technology and written informed consent was obtained from all participants. The PCOS patients of this study were diagnosed according to the Rotterdam standard. At least two of the following criteria should be met for a definite diagnosis of PCOS: menstrual irregularity (defined as less than eight menstrual cycles per year, or the absence of menstruation for 35 days or more); clinical hyperandrogenism (defined as a modified Ferriman–Gallwey score of over 6, or androgenic alopecia, or both); biochemical hyperandrogenism (defined as a testosterone level of over 2.81 nmol/l, or an androstenedione level of over 10.8 nmol/L, or both, which were the 95th percentile of the normal range for the population of this study); polycystic ovary morphology (defined as 12 or more follicles of 2–9 mm in diameter, or an ovarian volume greater than 10 cm3 in at least one ovary. In the control group, women of normal childbearing age who were treated in our hospital for azoospermia or asthenospermia were selected. They were planning to undergo artificial insemination with donor sperm, had regular menstrual periods, had not been menopausal, had no history of adverse pregnancy and childbirth. They had normal biochemical indexes, no polycystic ovary under ultrasound, no other Kaohsiung diseases such as congenital adrenal hyperplasia (CAH), Cushing syndrome, androgen-secreting tumors, and no endocrine diseases, no metabolic diseases, and family history.

In this study, blood samples from the subjects were collected in the morning on the third day of the menstrual cycle after at least 8 h of fasting. Upon collection, all blood samples were centrifuged immediately. The serum was separated and frozen at −80°C. Fasting plasma glucose was measured using a finger stick blood glucose method (Olympus, Japan). The levels of fasting insulin, testosterone, androstenedione, AMH, FSH, LH, and thyroid stimulating hormone were measured by chemiluminescence assays on an Immulite 2000 instrument (AXSYM, USA). For all measurements, the inter-assay coefficient of variation and the intra-assay coefficient of variation were less than 10% and 15%, respectively. Finally, 6 serum samples (3 PCOS samples and 3 normal subject samples) were selected for lncRNA sequencing, The 3 PCOS patients whose sisters were also PCOS patients were for the first-time confirmed PCOS and had not started drug treatment. The other 116 samples (60 PCOS samples and 56 normal subject samples) were subjected to evaluation by qRT-PCR. The clinical characteristics of the PCOS and normal subjects are listed in **Table 1**.

**Table 1:** The clinical characteristics of PCOS patients and normal subjects.

	PCOS (n=63) (mean±SD)	Normal (n=59) (mean±SD)	p-value
Age(years)	28.51 ± 5.94	31.56 ± 5.94	0.231
BMI(kg/m2)	24.01 ± 3.95	21.15 ± 2.86	0.039
T(ng/ml)	1.70 ± 0.66	0.99 ± 0.44	0.000
E2 (pg/ml)	160.36 ± 62.16	149.92 ± 56.07	0.075
P(ng/ml)	1.06 ± 1.25	1.25 ± 0.64	0.034
FSH (IU/L)	5.33 ± 1.18	5.60 ± 1.59	0.302
LH (IU/L)	6.65 ± 4.33	3.68 ± 1.73	0.000
LH/FSH	1.25 ± 0.71	0.72 ± 0.42	0.001
AMH(pmol/L)	10.98 ± 5.68	4.58 ± 3.30	0.000
INHB(pg/ml)	114.13 ± 49.30	115.37 ± 50.57	0.878
HOMA-IR	4.69 ± 2.48	1.84 ± 0.98	0.000

### RNA Extraction and Real-Time Quantitative PCR

Total RNA was extracted using the TRIzol reagent (Invitrogen, Carlsbad, CA, USA). RNA purity and integrity were assessed using an ND-1000 Nanodrop Instrument and an Agilent 2200 TapeStation, respectively. An NEBNext^®^ UltraTM Directional RNA Library Prep Kit (New England Biolabs, Ipswich, Massachusetts, USA) was used for the preparation of a RNA-seq library. Finally, the sequencing of libraries was conducted on an Illumina HiSeq™ 3000 system.

A computational pipeline was used to process RNA-seq data, which were mapped to human reference genome hg19 using TopHat v2.0.13 in conjunction with default parameters. Gfold V1.1.2 was subsequently employed to convert aligned short reads into read counts for each gene model in refseq. Differential expression was assessed by the AudicS method. The Benjamini–Hochberg multiple test correction method was enabled during the analysis. Subsequently, differentially expressed genes were chosen according to the criteria of fold change > 2 and adjusted q-value < 0.05. Finally, qRT-PCR was performed using the SYBR Select Master Mix (TaKaRa, Japan). During qRT-PCR, glyceraldehyde 3-phosphate dehydrogenase (GAPDH) was used as the internal control. All primer sequences used in qRT-PCR are shown in **Table 2**.

**Table 2:** The primer sequences of qRT-PCR are shown below.

Name	Primer
GAPDH	F: 5′-AGGGCTGCTTTTAACTCTGGT-3′ R: 5′-CCCCACTTGATTTTGGAGGGA-3′
ENST00000584923.1	F: 5′- CTTGGCATGTCGCGAGAAAG -3′ R: 5′- AATAGGAGGTGCCACACAGC -3′
ENST00000565493.1	F: 5′-ATGGAAAGAGGTTGCCGACG -3′ R: 5′- GGACGTATCGCTTCCAGAGG -3′
ENST00000508174.1	F: 5′- AAAGTTACGGAGGACCCAGC -3′ R: 5′- GACGGAGGTTGGAATGTGGA -3′
XR_245194	F: 5′-GCAAGGGTATGAACAAAGCG-3′ R: 5′-GCAGGTCTGGAAGGCACAA-3′
NR_002756	F: 5′-AATCTTTCGCCTTTTACTAA-3′ R: 5′-AAAATTGGGTTAAGACTCAG-3′

### Bioinformatics Analysis

All differentially expressed mRNAs were selected for GO and KEGG pathway analyses to investigate the potential role of lncRNAs co-expressed with these mRNAs. Performed using KOBAS2.0 software, GO analysis provides label classification of gene functions and gene product attributes (http://www.geneontology.org). In addition, GO analysis covers three domains: cellular component (CC), molecular function (MF) and biological process (BP). The false discovery rate (FDR) was used to denote the significance of the P-value (a FDR value of < 0.05 was recommended). The differentially expressed mRNAs and their enrichment in different pathways were mapped using the KOBAS2.0 software (http://www.genome.jp/kegg). The importance of the KEGG pathways among differentially expressed genes was denoted by the FDR value (a FDR value of < 0.05 was recommended).

### The Network of lncRNA and mRNA Co-Expression

To find the key lncRNAs involved in PCOS and their potential functions, a lncRNA/mRNA co-expression network was constructed to investigate the potential interactions between lncRNAs and mRNAs. During the construction of the lncRNA/mRNA co-expression network, Pearson correlation calculation was utilized to determine the normalized signal intensity of differentially expressed lncRNAs and mRNAs, while significantly correlated mRNAs with an absolute Pearson correlation coefficient > 0.99 were chosen as the targets to build the network using Cytoscape (version 3.5.0). Subsequently, six mRNA transcripts were selected using COR > 0.85 and P < 0.05, while the enrichment of these mRNAs in the chemokine signaling pathway was investigated in the following study. During the analysis, the target genes of all lncRNAs were predicted through cis- and trans-actions. For genes encoding several transcripts, the median value of different transcripts was taken as the value of the gene expression.

### Statistical Analysis

The expression levels of all lncrna and mRNA transcripts were analyzed by balltown software. The difference analysis parameters were Q-value < 0.05 and | log2 fold change | ≥ 1.The pathways with p-value < 0.05 were defined as those significantly enriched in differentially expressed genes, Pearson correlation coefficient was calculated in the construction of coexpression network, and the mRNA with correlation coefficient > 0.99 was selected as the construction target.

## Results

### Overview of lncRNA and mRNA Profiles

**Figure 1:**
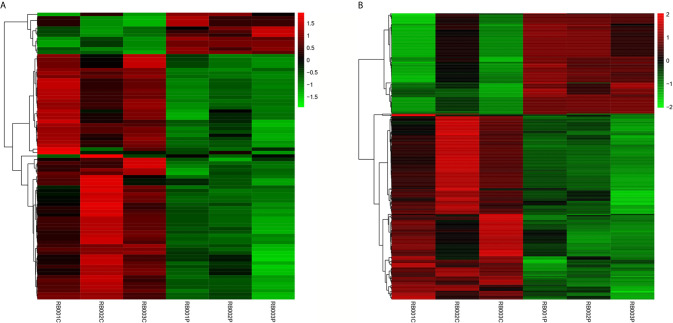
Expression profiles of differentially expressed lncRNAs and mRNAs in PCOS patients and normal subjects. The hierarchical cluster analysis generated heat maps of differentially expressed lncRNAs and mRNAs (log2 fold change > 2; q < 0.05). Color represents the log10 (RPKM + 1) value, with red indicating upregulated genes and green indicating downregulated genes. **(A)** lncRNA; **(B)** mRNA.

All RNA samples were subjected to high-throughput sequencing analysis of lncRNA and mRNA expression. In total, thousands of differentially expressed human lncRNAs and mRNAs were evaluated. The differentially expressed lncRNAs and mRNAs were selected when the fold change of expression was > 2.0 (q < 0.05). During the analysis, a positive value indicated up-regulation, while a negative value indicated down-regulation. Subsequently, the expression levels of lncRNAs and mRNAs in the peripheral blood samples isolated from three PCOS patients and three normal subjects were compared. In this study, 21,566 lncRNAs, 57,567 mRNA and 34,948 coding transcripts were examined in the peripheral blood samples isolated from PCOS patients and normal subjects. As a result, the expression of 4,048 lncRNAs (64 up-regulated, 3984 down-regulated) and 14,276 mRNAs (748 up-regulated, 13,528 down-regulated) was found to be significantly changed. The top 10 most differentially expressed (5 up-regulated and 5 down-regulated) mRNAs/lncRNAs are shown in **Tables 3** and **4**. In addition, heat maps of differentially expressed lncRNA and mRNAs were created for better illustration (**Figures 1A, B**).

**Table 3:** The top 5 differentially expressed mRNAs.

mRNA name	Gene name	Log2 (fold change)	Regulated
NM_000559.2	HBG1	7.87	Up
NM_001003938.3	HBM	3.11	Up
NM_000517.4	HBA2	2.86	Up
NM_001002841.1	MYL4	2.67	Up
XM_005257392.1	MYL4	2.67	Up
XM_005272808.1	HLA-DRA	-7.09	Down
XM_005270393.1	IFI44L	-4.14	Down
XM_005270392.1	IFI44L	-4.10	Down
NM_006820.2	IFI44L	-4.05	Down
XM_005270391.1	IFI44L	-4.04	Down

**Table 4:** The top 10 differentially expressed lncRNAs.

lncRNA name	Gene name	Log2 (fold change)	Regulated
NR_004391.1	RNY1	3.31	Up
NR_004393.1	RNY4	3.06	Up
NR_015424.1	ANKRD36BP2	2.78	Up
NR_003287.2	RNA28S5	2.78	Up
XR_247026.1	GUK1	2.22	Up
XR_246226.1	IFI44	-3.19	Down
NR_045117.1	DDX11L10	-2.94	Down
ENST00000560769.1	ENSG00000259279.1	-2.65	Down
NR_046396.1	XAF1	-2.49	Down
NR_046397.1	XAF1	-2.48	Down

### Examination of the Functions of Differentially Expressed mRNAs

**Figure 2:**
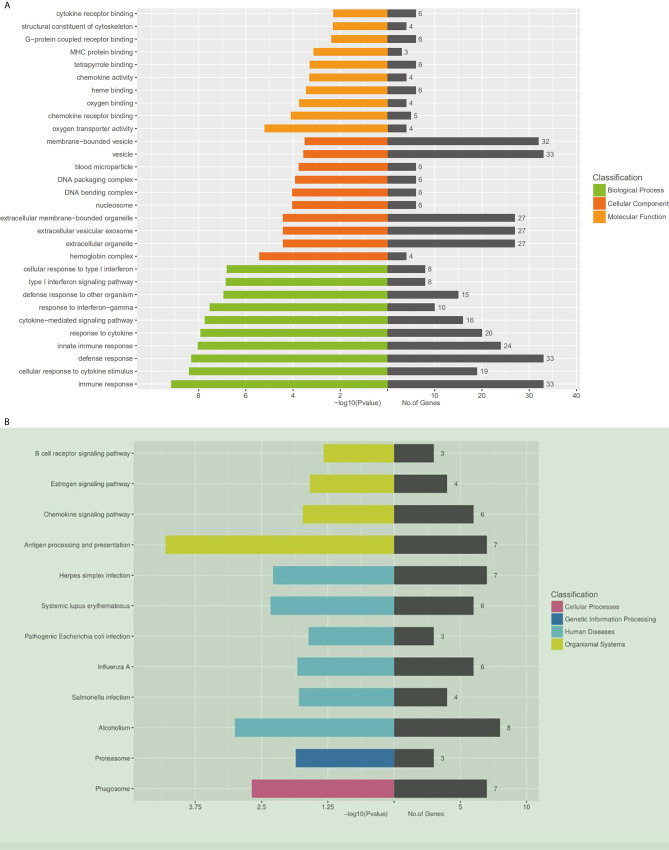
Gene ontology and KEGG pathway analysis of differentially expressed mRNAs. **(A)** Gene ontology. A total of 409 differentially expressed mRNAs are chosen in GO analysis, which are divided into three categories: the green columns represent the top 10 biological processes, the red represents the top 10 cellular components, and the orange represents the top 10 molecular functions. On the right side of the coordinate axis, the gray color represents the number of genes enriched in the pathway. **(B)** The KEGG pathway enrichment analysis was performed on the KEGG biological pathways database (http://www.genome.jp). The left side of the coordinate axis shows the P-values of the pathway, and the yellow, green, blue and purple colors indicate the KEGG classification: organismal systems, human diseases, genetic information processing and cellular processes. On the right side of the coordinate axis, the gray color represents the number of genes enriched in the pathway.

Using DAVID (The Database for Annotation, Visualization and Integrated Discovery), gene ontology (GO) and KEGG pathway analyses were conducted to understand the functions of the 14,276 differentially expressed mRNAs. The KEGG results showed that the differentially expressed genes were enriched in the B cell receptor signaling pathway, estrogen signaling pathway, and chemokine signaling pathway. Moreover, the genes were related to the expression of proteasome and phagosome (**Figure 2A**). In addition, the GO results showed that these genes may be involved in the following cellular processes: cytokine receptor binding, MHC protein binding, chemokine activity oxygen binding, and chemokine receptor binding (**Figure 2B**). All these results pointed to an inflammatory state of the organism.

### Construction of an lncRNA and mRNA Co-Expression Network

**Figure 3:**
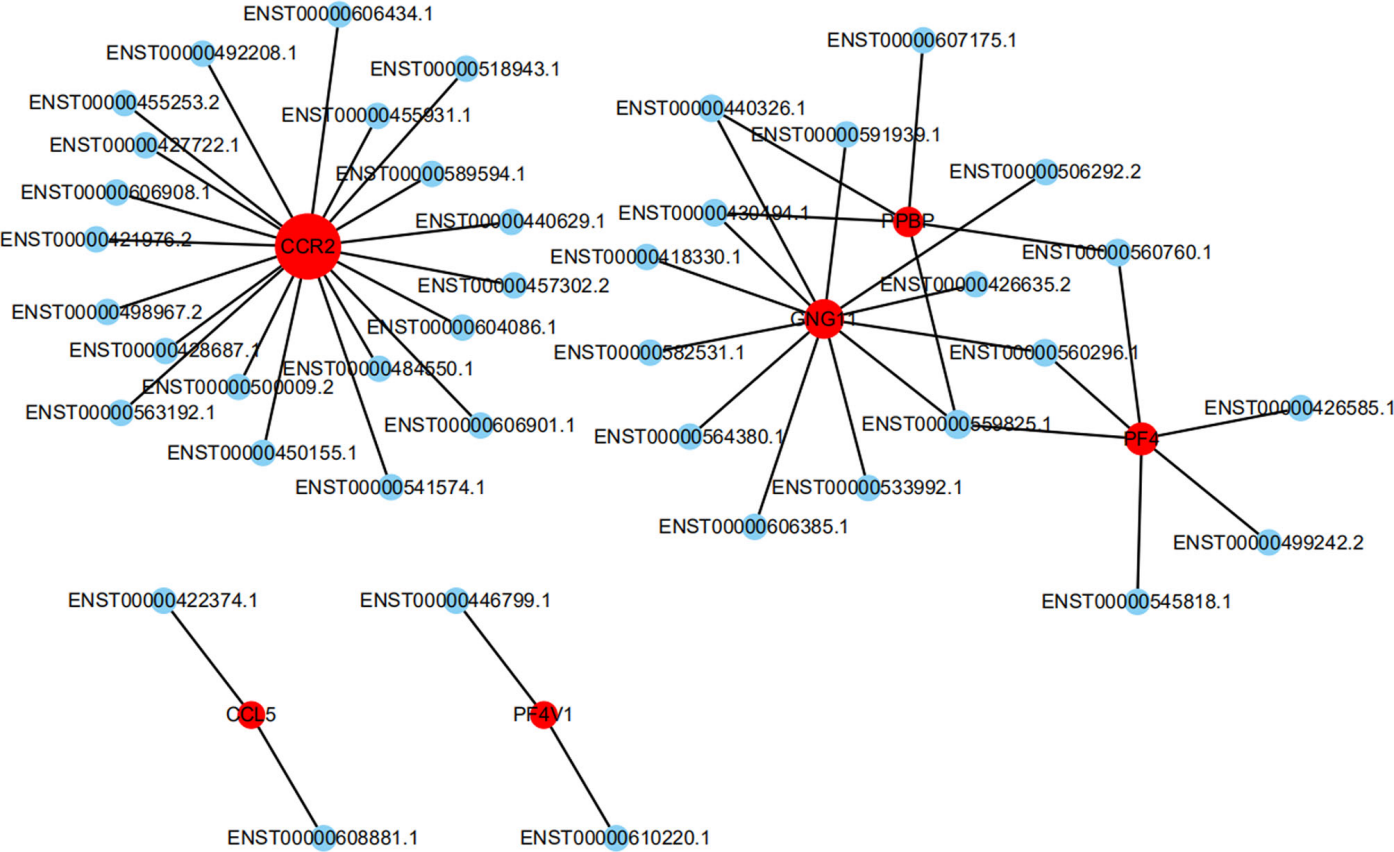
The network represents the co-expression correlations between the significantly differentially expressed mRNA and lncRNA transcripts in the chemokine signaling pathway. Red circles indicate mRNA transcripts, and blue circles indicate lncRNA. Solid lines indicate correlations.

To further investigate the inflammatory state of PCOS, chemokines were chosen for deep-going analysis using an lncRNA-mRNA co-expression network. The chemokine signaling pathway involved 6 mRNAs and 41 lncRNAs. Among these lncRNAs, 20 lncRNAs may be involved in the regulation of CCR2 expression. ENST00000484550.1 (lincRNA, -2.0 fold change), and ENST00000492208.1 (lincRNA, -2.2 fold change), ENST00000606434.1 (lincRNA, -2.5 fold change), may be greatly involved in the regulation of CCR2 expression (**Figure 3**).

### Validation by Quantitative RT-PCR

**Figure 4:**
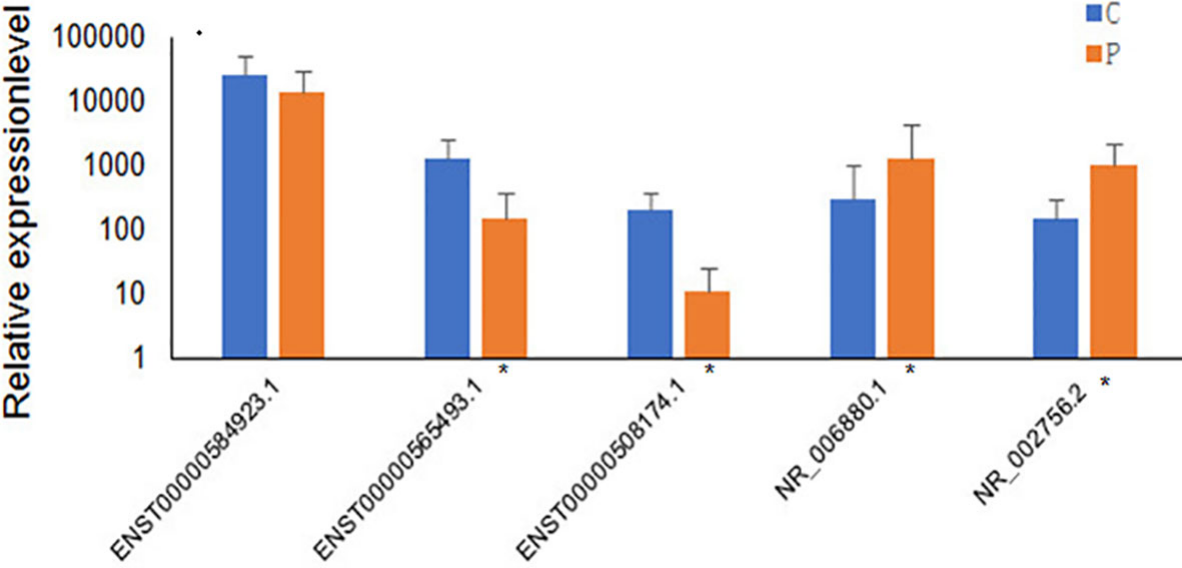
Validation of high-throughput sequencing results using qRT-PCR analysis of 5 randomly selected lncRNAs. The blue columns refer to the control group(n=60), and orange refers to PCOS group (n=56). Groups with significant differences are marked with *P < 0.05.

Several differentially expressed genes were randomly selected and further validated by qRT-PCR. The five lncRNAs selected for qRT-PCR validation were ENST00000584923.1, ENST00000565493.1, ENST00000508174.1, NR_006880.1, and NR_002756.2. The qRT-PCR results suggested that the fold changes of these lncRNAs observed in the high-throughput sequencing analysis were correct (**Figure 4**).

## Discussion

PCOS is the most common endocrine abnormality in women of reproductive age, yet its etiology remains unknown. It has been reported that lncRNAs play important roles in a wide range of functional activities. In recent years, our team has tried to understand PCOS from a genetic perspective by establishing a stable PCOS animal model using gene knock-out. In addition, the expression of some miRNAs in peripheral blood of PCOS patients was analyzed. More and more studies have focused on the role of lncRNA in follicular maturation. The proliferation, differentiation and apoptosis of granulosa cells determine the quality of follicular development. Research data show that a variety of lncRNAs can affect the development of granulosa cells. Liu et al. (14) reported for the first time that lncRNA HCG26 level was up-regulated in PCOS patients, while lncRNA HCG26 knockdown inhibited the proliferation of granulosa cells. Li et al. (15) confirmed that lncRNA steroid receptor RNA agonist (lncRNA SRA) can stimulate the growth of mouse granulosa cells *in vitro*. Huang et al. (9) confirmed for the first time the whole genome lncRNA expression pattern in cumulus cells of PCOS patients through microarray, and revealed that the differentially expressed lncRNA affects the development of oocytes. Nevertheless, few studies have focused on lncRNA-mRNA co-expression in PCOS, while the molecular mechanisms underlying PCOS remain poorly understood. Therefore, an integrated analysis of differentially expressed lncRNAs and mRNAs in PCOS could help to understand its pathogenesis.

Using the novel technology of RNA-seq analysis, it was demonstrated in this study that the expression of lncRNAs and mRNAs in PCOS patients was different from that in normal subjects. In total 4,048 lncRNAs (64 were up-regulated and 3,984 were down-regulated) and 14,276 mRNAs (748 were up-regulated and 13,528 were down-regulated) were differentially expressed between PCOS patients and normal subjects. These results were quite different from Jiao’s (16). previous study which found a total of 1583 new lncRNAs were differentially expressed in follicular fluid of healthy women and PCOS patients, such a discrepancy may be due to the differences in samples and tissues. In addition, it is believed that PCOS is not only limited to local ovarian lesions, but also affects multiple systems. Therefore, the blood specimens can more comprehensively reflect the status of PCOS, while the local microenvironment and local cytokines in the ovary may play a more limited role in the pathogenesis of PCOS.

Through the bioinformatics analysis conducted in this study, it was found that a large number of differentially expressed lncRNAs and mRNAs were involved in the onset of a chronic inflammatory state in PCOS. Previous studies have shown the role of a low-grade and chronic inflammation in the development of PCOS (17) and its long-term complications. In addition, as small proteins that can activate leukocytes during the process of inflammation, chemokines such as MCP-1, IL-8, CXCR2, IL-6 and TNF have been implicated in PCOS (18, 19). Since chemokines are involved in the state of low-grade inflammation, the alternation of chemokine signaling pathway was studied here to understand its role in the pathogenesis of PCOS from a genetic perspective.

The results of co-expression analysis conducted in this study were based on the expression of lncRNAs and mRNAs during PCOS. As lncRNAs do not encode proteins, the annotation for their biological functions needs to be interpreted in other ways. For each differentially expressed lncRNA, its co-expressed coding genes were screened to find correlated RNA-mRNA pairs, thus deducing the function of lncRNAs from that of corresponding mRNAs. Based on the results of high-throughput sequencing of the chemokine signaling pathway, 63 lncRNAs were found to interact with 6 mRNAs. Among these lncRNAs, 20 lncRNAs may be involved in the regulation of CCR2 expression. Since lncRNAs are often named according to their locations in the genome relative to the location of protein coding genes, lncRNA genes located between two protein coding genes are often referred to as long or large intergenic or intervening lncRNAs (lincRNAs). In order to facilitate the follow-up experiments, the relevant lincRNAs, such as ENST00000457302.2, ENST00000484550.1, ENST00000492208.1, and ENST00000606434.1, were selected and verified.

To the knowledge of the authors, this is the first lncRNA-mRNA co-expression analysis conducted using the peripheral blood samples from PCOS patients. Notably, the co-expression network of coding-non-coding genes provided valuable insight regarding the pathogenesis of PCOS. Nowadays, the number of identified lncRNAs is growing quickly and hence further study will be needed to explore their molecular and biological functions. There are limitations to this study. First, the complex blood background made it difficult to identify the cellular origin and tissue expression patterns of lncRNAs among peripheral blood samples of PCOS patients. Second, the sample size was small and contained only 63 PCOS patients and 59 healthy controls. Third, the results were obtained from the bioinformatic analysis and high-throughput sequencing analysis only. Therefore, further studies are needed to confirm these differentially expressed genes and their roles in different pathways.

## Data Availability Statement

The original contributions presented in the study are included in the article/supplementary material. Further inquiries can be directed to the corresponding author.

## Ethics Statement

The studies involving human participants were reviewed and approved by Ethics Committee of Guangdong Institute of family planning science and technology. The patients/participants provided their written informed consent to participate in this study.

## Author Contributions

All authors contributed to the article and approved the submitted version.

## Funding

This work was supported in part by Technology Planning Project of Guangdong Province (grant numbers: 2013B032000001; 2014A020212229), the Guangdong Natural Science Foundation (grant numbers: 10151063201000036; S2011010002526), and Guangzhou Science and Technology Plan Project (grant number: 201804010003).

## Conflict of Interest

The authors declare that the research was conducted in the absence of any commercial or financial relationships that could be construed as a potential conflict of interest.
